# Gp130 Promotes Inflammation via the STAT3/JAK2 Pathway in Allergic Conjunctivitis

**DOI:** 10.1167/iovs.64.4.5

**Published:** 2023-04-06

**Authors:** Jiayu Bao, Peng Zhang, Binge Wu, Jingyi Wang, Siyuan Li, Ao Li, Ying Jie

**Affiliations:** 1 Beijing Institute of Ophthalmology, Beijing TongRen Eye Center, Beijing Key Laboratory of Ophthalmology and Visual Sciences, Beijing Tongren Hospital, Capital Medical University, Beijing, China; 2 Department of Ophthalmology, the Second Affiliated Hospital of Baotou Medical College, Inner Mongolia University of Science and Technology, Inner Mongolia, China

**Keywords:** allergic conjunctivitis, gp130, inflammation, Janus kinase 2, STAT3 protein

## Abstract

**Purpose:**

Allergic conjunctivitis (AC) is a common allergic condition worldwide that requires accurate screening and early diagnosis. We found that gp130 is essential for AC, as gp130 levels are elevated in AC. Therefore, this study aimed to elucidate the functions and the possible underlying mechanisms of gp130 in AC.

**Methods:**

To compare mRNA expression profiles, the conjunctival tissues of BALB/c mice with ovalbumin (OVA)-induced AC were subjected to RNA-sequencing (RNA-seq) analysis followed by bioinformatic analysis. A nonrandomized study involving 57 patients with AC and 24 sex- and age-matched healthy individuals was conducted. A protein chip was used to detect cytokine levels in patient tears. Differentially expressed proteins in patient serum were detected using label-free quantitative mass spectrometry. Histamine-stimulated conjunctival epithelial cells (HConEpiCs) were used to construct a cell model. LMT-28 which can inhibit gp130 phosphorylation was dropped onto the murine ocular surface, and the resulting symptoms were observed.

**Results:**

Gp130 is upregulated in the conjunctival tissues of OVA-induced mice, the serum and tears of patients, and the histamine-stimulated HConEpiCs. Signal transducer and activator of transcription 3 (STAT3) and Janus kinase 2 (JAK2) were upregulated in the conjunctival tissues of mice with OVA-induced AC and in HConEpiCs. Ocular surface inflammation was significantly relieved in LMT-28-treated mice. The expression of IgE, IL-4, IL-5, and IL-13 in serum of LMT-28-treated mice decreased. The number of mast cells in conjunctival tissue was decreased compared with OVA-induced mice.

**Conclusions:**

Gp130 may play an important role in AC via the gp130/JAK2/STAT3 pathway. Inhibiting gp130 phosphorylation alleviates ocular surface inflammation in mice, presenting a potential treatment approach for AC.

Allergic conjunctivitis (AC) is the most common allergic condition worldwide. Climate change, pollution, and increased pollen loads have resulted in increased AC incidence and heightened immunological sensitivity among individuals.^1^ AC symptoms include chemosis, tearing, increased tear mucus, and itching. These symptoms can pose a threat to human health and considerably impact work and life.^2^ The increasing AC prevalence exacerbates physiological problems, contributing to significant health, economic, and social burdens.^3^ Hence, the mechanisms underlying the pathological transformation and pathogenesis of AC warrant further elucidation.

A variety of tissues and cell types are affected by cytokines and their receptors.^4^ Several cytokines are clinically useful in AC. When mucosal tissue becomes inflamed, eosinophil granule proteins are released, resulting in epithelial damage and subepithelial injury.^5^^,^^6^ IL-4 and CCL17/TARC are biomarkers of Th2-type inflammation,^7^^,^^8^ and eotaxin, TNF-α, and sIL-6 receptor expression is abnormal in giant papillae.^9^^–^^11^ Discovering suitable cytokines and cytokine receptors is necessary to gain greater insight into AC immunology and pathophysiology. We hypothesized that many cytokines are dysregulated in AC progression. To investigate the mechanism underlying the associations between cytokines and AC, we screened cytokines that are dysregulated in AC and found that glycoprotein 130 (gp130) was upregulated in AC and functionally aggravated inflammation during AC.

Gp130 is a highly conserved, 130-kDa transmembrane protein in vertebrates.^12^ Several studies over the past 20 years have examined gp130. In addition to being a ubiquitous signal-transducing receptor, gp130 belongs to a part of the receptor complex for several cytokines, such as IL-11 and IL-6.^13^^,^^14^ Gp130 expression and activation regulate various biological processes, including inflammation and immune responses.^15^^,^^16^ Type II cytokine production and innate lymphoid cell type 2 (ILC2) differentiation can be inhibited by IL-35 via gp130 regulation. Thus, gp130 inhibits the ILC2 response in allergic asthma.^17^ A designer fusion protein (gp130-Fc) can antagonize sIL-6R-mediated trans-signaling by IL-6R in lung tissues. After ovalbumin (OVA) peptide sensitization and challenge, gp130 can also transduce IL-6R signaling via an antibody that targets the IL-6R gp80 unit (alphaIL-6R).^18^ However, no previous studies have investigated the relationship between gp130 and AC. LMT-28 is the first synthesized IL-6 pathway inhibitor that functions as direct binding to gp130. LMT-28 shows low toxicity and selectively inhibits IL-6 activated phosphorylation of STAT3, JAK2, and gp130.^19^ Therefore, we use LMT-28 as an antagonist to study the mechanistic role of gp130 in AC.

## Methods

### Animal Model

Forty-five 6 to 8-week-old male BALB/c mice were used. A slip lamp was used to assess the eyes of each mouse before treatment to confirm normal physiology, and the mice were divided into three groups: OVA (OVA, *n* = 15), control (NC, *n* = 15), and treatment (LMT-28, *n* = 15). On days 0 and 7, 5 g of OVA and 15 mg/mL aluminum hydroxide adjuvant were injected subcutaneously into OVA-immunized mice in 200 µL of sterile saline.^20^^–^^22^ Two weeks after immunization, the mice were challenged with topical OVA eye drops (250 mg in phosphate-buffered saline [PBS]) once per day for a minimum of 9 days. Control animals received PBS. Treatment animals received OVA and LMT-28 (LMT-28 is dissolved in DMSO [10 mM/mL]) and further prepared in PBS (1 ug/uL) as a working solution. LMT-28 was applied to the eye surface for 30 minutes after OVA stimulation (3 times/day). The eyes and eyelids were collected after the mice were euthanized. First, the eyeballs with both eyelids were harvested surgically under dissecting microscope. After that, both palpebral and bulbar conjunctiva were separated and collected. Conjunctival tissues were preserved for future use. All animal experiments were performed according to the guideline and obtained ethical permission from the animal research ethics committee.^23^ All animal experiments have complied with the Association for Research in Vision and Ophthalmology Statement for the Use of Animals in Ophthalmic and Vision Research.

### RNA Extraction and Quantitative Real-Time Polymerase Chain Reaction

TRIzol (Yeasen Biotech, Shanghai, China) was used to extract total RNA. Total RNA was reverse transcribed into cDNA by cDNA Synthesis Super Mix. Subsequently, gene expression was measured using quantitative real-time polymerase chain reaction (qRT‒PCR) with SYBR Green Master Mix. Glyceraldehyde 3-phosphate dehydrogenase (GAPDH): forward primer: 5′-ACCACAGTCCATGCCATCAC-3′; reverse primer: 5′-TCCACCACCCTGTTGCTGTA-3′; and Gp130: forward primer: 5′-GCGTACCTCAAACAAGCC-3′; and reverse primer: 5′- TGAAGCCATTCTGGTCGT-3′.

### RNA-Sequencing and Analysis

On day 22, each conjunctiva was isolated, and RNA was extracted as described above as RNA Extraction and Quantitative Real-Time Polymerase Chain Reaction. RNA libraries were prepared using the NEBNext Ultra RNA Library Prep Kit for Illumina (New England Biolabs) and sequenced on an Illumina NovaSeq 6000 platform (Echo Biotech Co., Ltd., Beijing, People's Republic of China). The reads generated by RNA-sequencing (RNA-seq) were aligned to the mouse reference genome GRCm38 using HISAT2. On the basis of Ensembl genome annotation (release 101), StringTie was applied to assign the reads to genes. R software was used to perform differentially expressed gene analysis (*P* * 0.01) using edgeR or EBSeq. Fisher's exact test was used to calculate *P* values.

### Human Participants

From May to June 2021, 57 patients (29 men and 28 women; mean age = 31 years; age range = 21–40 years; about 30% of patients have a family history of AC) with AC were enrolled in the study. The hospital ethics committees provided ethics approval (Ethics number: HX-015) for the study. Before participating in the study, all subjects provided informed written consent. A healthy control group of 24 sex- and age-matched subjects (12 men and 12 women; mean age = 32 years; age range = 21–40 years) without a history of allergic diseases was also recruited for the study. The clinical trial registry information was as follows: progress and outcome of allergic conjunctivitis: a prospective cohort study, World Health Organization International Clinical Trials Registry Platform, Chinese Clinical Registry, and Clinical Trials (Registration number: ChiCTR2000040691). Tear fluid was collected with capillaries from the conjunctival sac of the patient, which did not stimulate the patient. The tears were collected in a sterile 1.5 mL centrifuge tube and stored in −80°C.^24^^–^^26^ Tears were collected at the initial and the following two follow-up visits. The patients did not receive any anti-allergic treatment when their tears were collected for the first time. All patients were treated with the same medication. The specific scoring standard of eye itch is 0 as none, 1 as intermittent itching sensation, 2 as continual awareness but without the desire to rub, 3 as continual awareness with the desire to rub the eyes, and 4 as subject insists on rubbing their eyes. The mild group was scores of 1 and 2 for itch and the severe group was scores of 3 and 4 for itch.^27^^–^^29^

### Inclusion and Exclusion Criteria

Exclusion criteria: (1) patients who suffered from dry eye, atopic conjunctivitis of the eyelids, vernal keratoconjunctivitis, or atopic keratoconjunctivitis; (2) patients who wore contact lenses, those who have had cataract and corneal refractive surgery or infectious conjunctivitis, as well as patients with other allergies; and (3) patients aged >60 years or <5 years. Inclusion criteria: (1) the patient experienced their first occurrence of AC in 2021 and had no previous history of allergic disease; (2) the patient had a positive serum total IgE test result; And (3) AC was diagnosed according to subjective and objective symptoms.^30^

### Label-Free Quantitative Mass Spectrometry

Blood samples from three patients and three healthy volunteers were collected randomly for serum isolation. The label-free quantitative mass spectrometry assay and data analysis were performed by Wayen Biotechnologies (Shanghai).

### Protein Array

Human Antibody Array 507 was used to screen the expression of proteins (H-Wayen Biotechnologies, Shanghai, China). Patients (*n* = 14) with AC were randomly selected from 57 patients, and 10 µL of tears were collected at initial diagnosis as well as at 2 and 4 weeks after the initial diagnosis. Tears (10 µL) were similarly collected from 14 healthy volunteers. The 14 samples from each group were mixed for further analysis.

### Measurement of gp130 Levels in Tears

Tears (10 µL) were collected from the remaining 40 patients during their first visit using a capillary pipette. A Luminex assay was performed by Seninda Biomedical Corporation, China to analyze gp130 levels in tears (Human Premixed Multi-Analyte Kit, Bio-Techne).

### Determination of Serum Total Immunoglobulin E and gp130 Levels

Blood (2 mL) was collected from the remaining 40 patients during their initial visit. After separation, the serum was frozen at −80°C for downstream analysis. IgE and gp130 concentrations were quantified via enzyme-linked immunosorbent assay (ELISA; IgE Human ELISA Kit, BMS2097, ThermoFisher, USA; sIL-6ST/gp130/CD130 Human ELISA Kit, EHIL6ST, ThermoFisher, USA).

### Immunohistochemistry and Histopathology

Whole eyes were removed from mice, immediately fixed in 4% paraformaldehyde (pH 7.4), and embedded in paraffin. Immunohistochemistry was performed according to a standard immunohistochemical technique. A gp130-specific rabbit polyclonal antibody (catalog number: abs131809, Absin), an IL-5-specific rabbit polyclonal antibody (catalog number: abs118045, Absin), and an IL-4-specific rabbit polyclonal antibody (catalog number: abs131221, Absin) were used. Toluidine blue staining was performed to observe mast cells in the conjunctiva.

### Western Blot Assays

The protein levels in samples were determined using Western blot assays. The following primary antibodies were used: anti-Gp130 (abs139867), anti-p-gp130 (abs131809), anti-IL-5 (abs118045), and anti-IL-4 (abs131221) obtained from Absin Bioscience (Pudong, Shanghai); and anti-Janus kinase 2 (JAK2, 3230), anti-p-JAK2 (3771), anti-signal transducer and activator of transcription 3 (STAT3, 9139T), and anti-p-STAT3 (9145T) obtained from Cell Signaling Technology. Anti-GAPDH (30202) and secondary antibodies were obtained from Yeasen Biotechnology (Shanghai, China).

### Cell Culture and Treatment

Human conjunctival epithelial cells (HConEpiCs) were obtained from the Beijing Key Laboratory of Ophthalmology & Visual Science and maintained in Dulbecco's modified Eagle's medium (DMEM) (11965092, Gibco) supplemented with 10% fetal bovine serum (Gibco). Humidified chambers with 5% CO_2_ were used to grow HConEpiCs. The control group was treated with 0.1% dimethyl sulfide (DMSO). Cells were stimulated with 0.1 µmol/L histamine for 24 hours.^31^^–^^33^ After different treatments, the HConEpiC supernatant was collected for ELISA analysis.

### Cell Viability Assay

Ninety-six-well plates were seeded with 5000 HConEpiCs and incubated for 24 hours. Afterward, they were incubated with various concentrations of histamine (0, 0.025, 0.05, or 0.1 µmol/L) for 24 hours. The cell viability of HConEpiCs was then detected using the 3-(4,5-dimethylthiazol-2-yl)-2,5-diphenyl-2H-tetrazolium bromide (MTT) assay.

### Measurement of the Levels of IgE, IL-4, IL-5, and IL-13 in the Serum of Mice

The concentration of total IgE was measured using a mouse IgE ELISA kit (EMIGHE, ThermoFisher, USA), according to the manufacturer's protocol. IL-4, IL-5, and IL-13 levels were also measured by the ELISA kits (IL-4: RAB0300, Sigma-Aldrich Shanghai Trading, China; IL-5: RAB0304, Sigma-Aldrich Shanghai Trading, China; IL-13: RAB0257, Sigma-Aldrich Shanghai Trading, China).

### Quantification and Statistical Analysis

Prism (GraphPad) was used to analyze and graph data. We present data as the mean ± standard deviation. Spearman's correlation coefficient was used to study any associations between variables. A two-tailed Mann‒Whitney *U* test and an unpaired two-tailed Student's *t*-test were used for comparisons, with significance defined as *P* < 0.05 (*), *P* < 0.01 (**), *P* < 0.001 (***), and *P* < 0.0001 (****).

## Results

### mRNA Expression Profiles, Protein Profiles, and Screening of Secreted Proteins in AC

**Figure 1.**
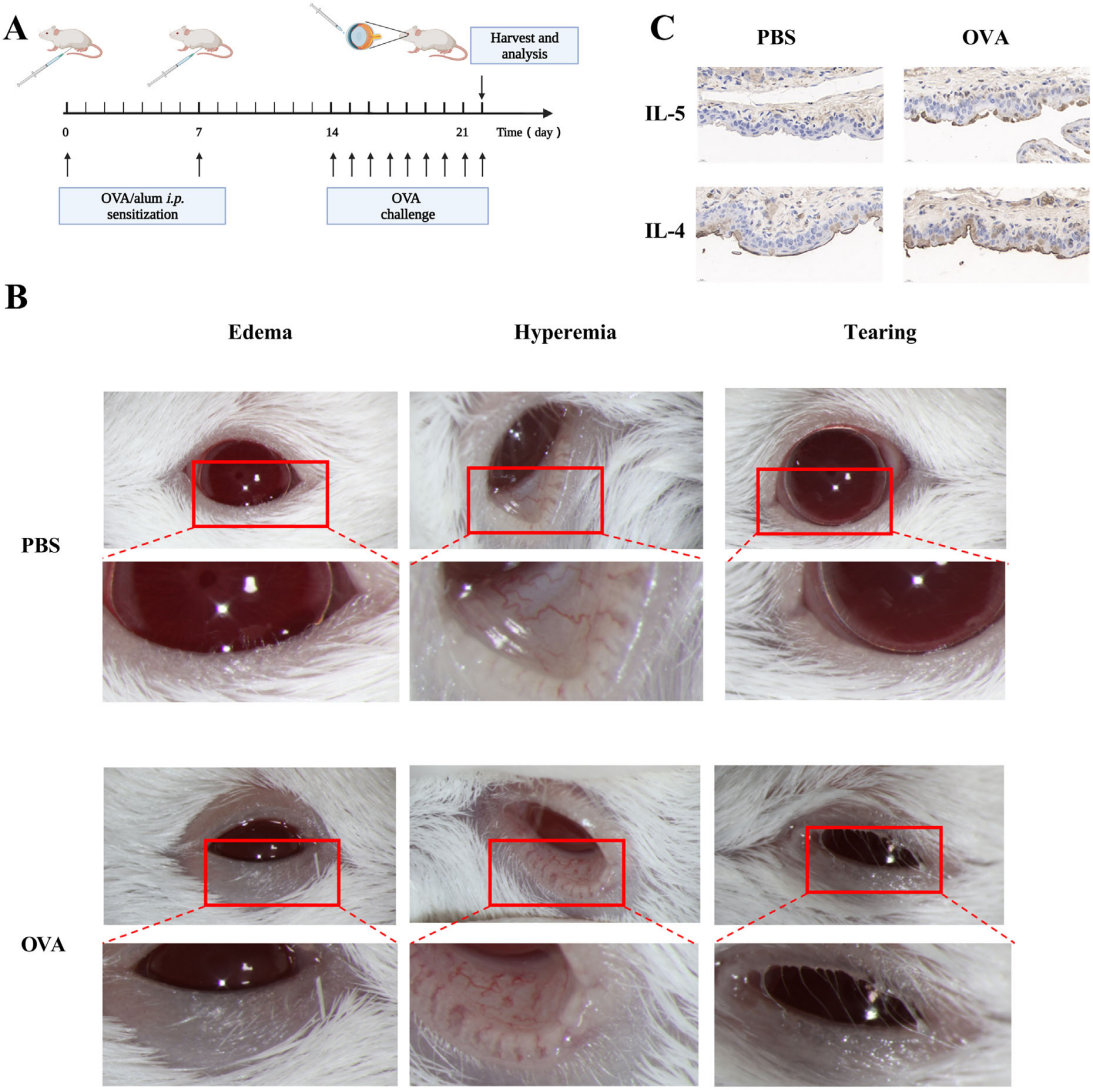
Construction of mouse model of allergic conjunctivitis (AC). (**A**) Procedure for generating ovalbumin (OVA)-induced AC in mice. (**B**) Ocular surface symptoms in mice. (**C**) IL-4 and IL-5 expression determined by immunohistochemical staining (80×). IL-4 and IL-5 are stained *light* or *dark brown*.

**Figure 2.**
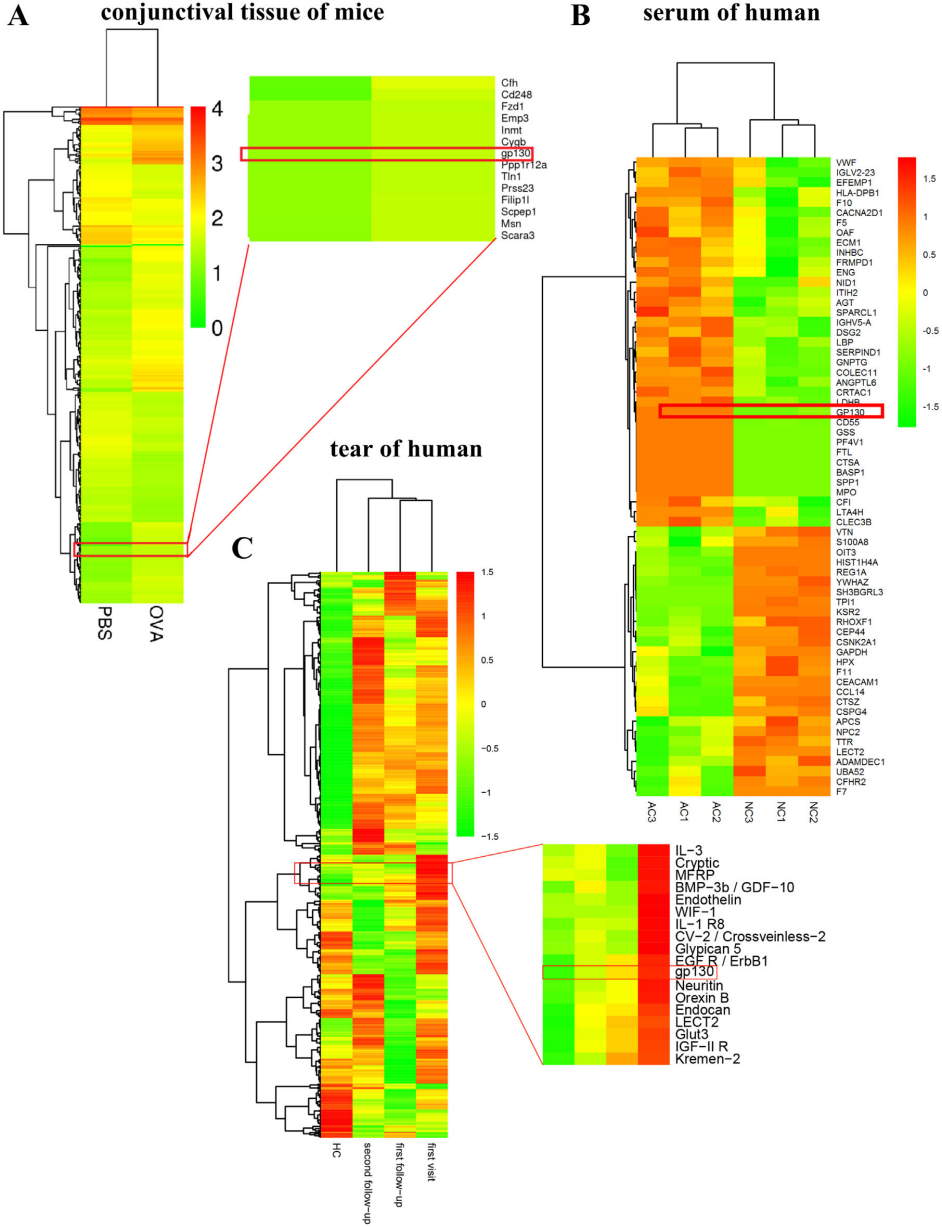
mRNA expression profiles, protein profiles, and screening of proteins in allergic conjunctivitis (AC). (**A**) Heatmap of differentially expressed mRNAs in normal conjunctival tissue and OVA-induced conjunctival tissue. Phosphate-buffered saline (PBS), normal group; OVA, OVA-induced mouse group. (**B**) Heatmap of differentially expressed proteins in patient serum and healthy volunteer serum. AC, allergic conjunctivitis patients; HC, healthy volunteers. (**C**) Heatmap of differentially expressed cytokines in tears.

To explore mRNA expression profiles in AC, we developed an OVA-induced mouse model to obtain AC conjunctival tissue (Fig. 1A). On day 14, PBS-challenged controls showed no notable abnormal ocular symptoms (Fig. 1B), whereas OVA-challenged mice exhibited changes indicative of AC development. Specifically, notable eyelid redness and swelling, conjunctival congestion and edema, and tearing were observed. Significant IL-4 and IL-5 protein level increases were confirmed using immunohistochemical analysis of conjunctival tissues (Fig. 1C). Serum IgE, IL-4, IL-5, and IL-13 level were determined in mice and the results displayed a high level of IgE, IL-4, IL-5, and IL-13 in OVA-challenged mice (Supplementary Fig. S1A). Inflammatory infiltration of mast cells was verified by toluidine blue staining (Supplementary Fig. S1B). These findings indicate that AC was successfully induced in the mice by OVA treatment. We then performed RNA-seq analysis of conjunctival tissues and corresponding normal mouse model tissues. The down- and upregulated genes are shown in a heatmap (Fig. 2A). The protein profile identified from the mass spectrum of a serum sample was composed of 64 proteins (Fig. 2B). To explore cytokine involvement in AC, the levels of inflammatory cytokines and inflammatory factors in tears were analyzed. Following remission, protein chip analysis revealed a decrease in inflammatory cytokines (Fig. 2C). Simultaneously, genes and differentially expressed genes present in the three assays were intersected to identify potential target genes for gp130.

### Gp130 is Upregulated in AC

**Figure 3.**
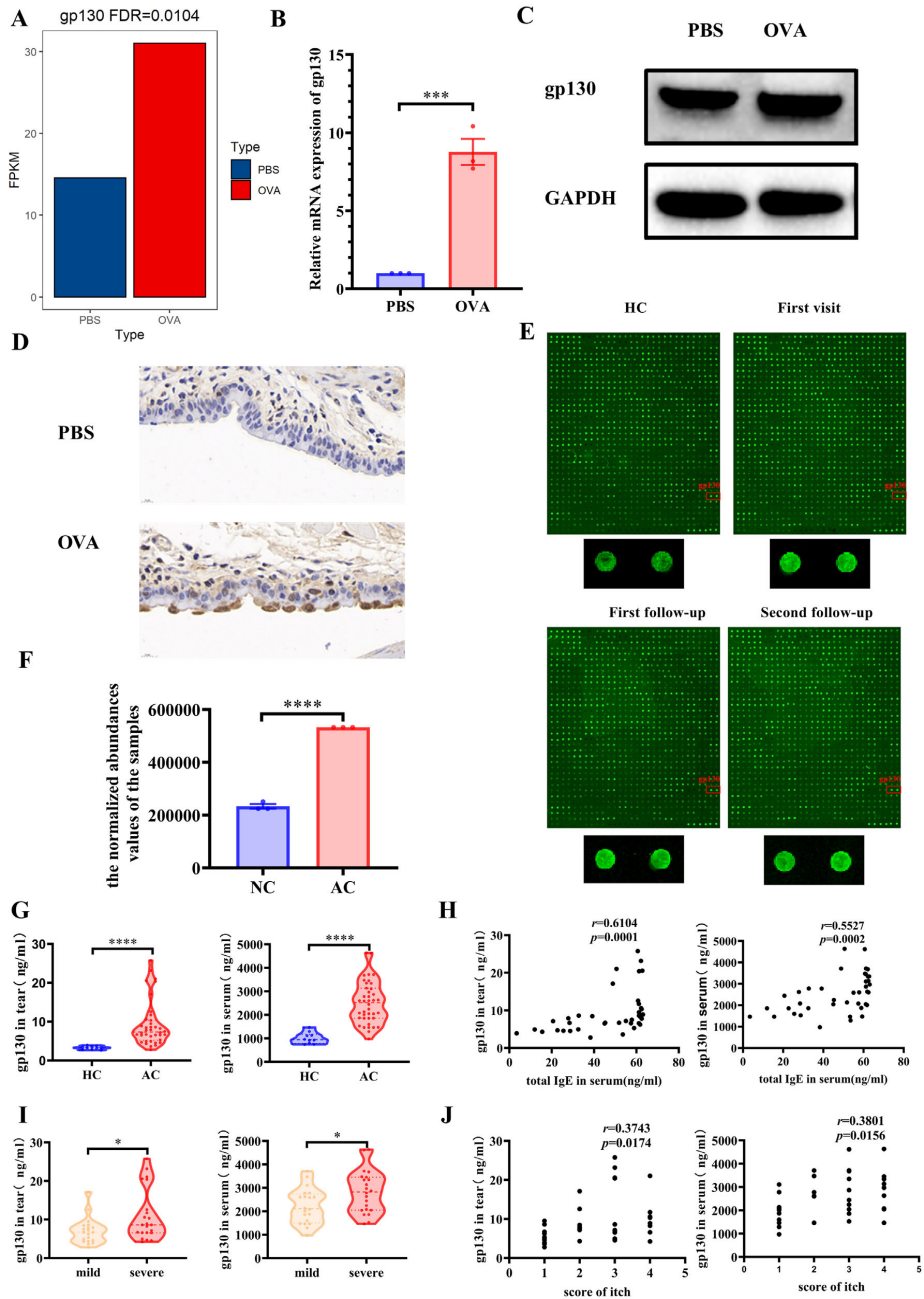
Gp130 is upregulated in allergic conjunctivitis (AC). (**A**) Boxplot of gp130 mRNA expression. (**B**) Quantitative real-time polymerase chain reaction (qRT‒PCR) analysis of gp130 expression in normal mouse conjunctival tissue (PBS; *n* = 3) and ovalbumin (OVA)-induced mouse conjunctival tissue (OVA; *n* = 3). (**C**) Protein levels of gp130 in mouse conjunctival tissue as detected using Western blotting with glyceraldehyde 3-phosphate dehydrogenase (GAPDH) as the loading control. (**D**) Gp130 expression determined by immunohistochemical staining (80×). Cells expressing gp130 are stained *light* or *dark brown*. (**E**) A protein array analysis of gp130 revealed significant changes in expression. HC, healthy volunteers. (**F**) Mass spectrometry results show considerable changes in the expression of gp130. NC, normal controls; AC, allergic conjunctivitis patients. (**G**) Baseline gp130 concentration levels in tears (*left*) and serum (*right*). (**H**) Correlations between immunoglobulin E concentrations and gp130 in tears (*left*) or serum (*right*). HC, healthy volunteers; AC, allergic conjunctivitis. (**I**) Difference in gp130 concentrations between the mild group (scores of 1 and 2 for itch) and the severe group (scores of 3 and 4 for itch). (**J**) Correlation between the gp130 concentration and itch scores. PBS, normal mouse group. OVA, ovalbumin-induced mouse group. *P < 0.05, ****P < 0.0001.

Based on the bioinformatics analysis, gp130 was selected, as this gene was upregulated in AC and closely associated with inflammation. In the murine model, RNA-seq analysis of OVA-induced mouse conjunctival tissue revealed high levels of gp130 expression (approximately 30 FPKM; Fig. 3A). The gp130 was upregulated in the conjunctival tissue of OVA-challenged mice (Fig. 3B). Impressively, Western blot (Fig. 3C) and immunohistochemistry results showed that, compared with normal mice, OVA-induced mice exhibited increased levels of gp130 in the conjunctiva (Fig. 3D). The results of a protein array showed that the expression of gp130 in the tears of patients with AC increased and then gradually decreased with disease remission (Fig. 3E). We also found high expression of gp130 in the mass spectrometry results for patient serum (Fig. 3F). Based on the strict screening criteria, 40 patients were selected to verify the results of the chip and mass spectrometry assays. Significant differences were observed in the levels of gp130 between patients with AC and control subjects for tears and serum (Fig. 3G). According to Spearman correlation analysis results, gp130 in serum and tears was positively correlated with serum total IgE (Fig. 3H). Significant positive correlations existed between serum and tear concentrations of gp130 and itch scores (Figs. 3I, 3J).

### Increased Expression of gp130 in Histamine-Stimulated HConEpiCs

**Figure 4.**
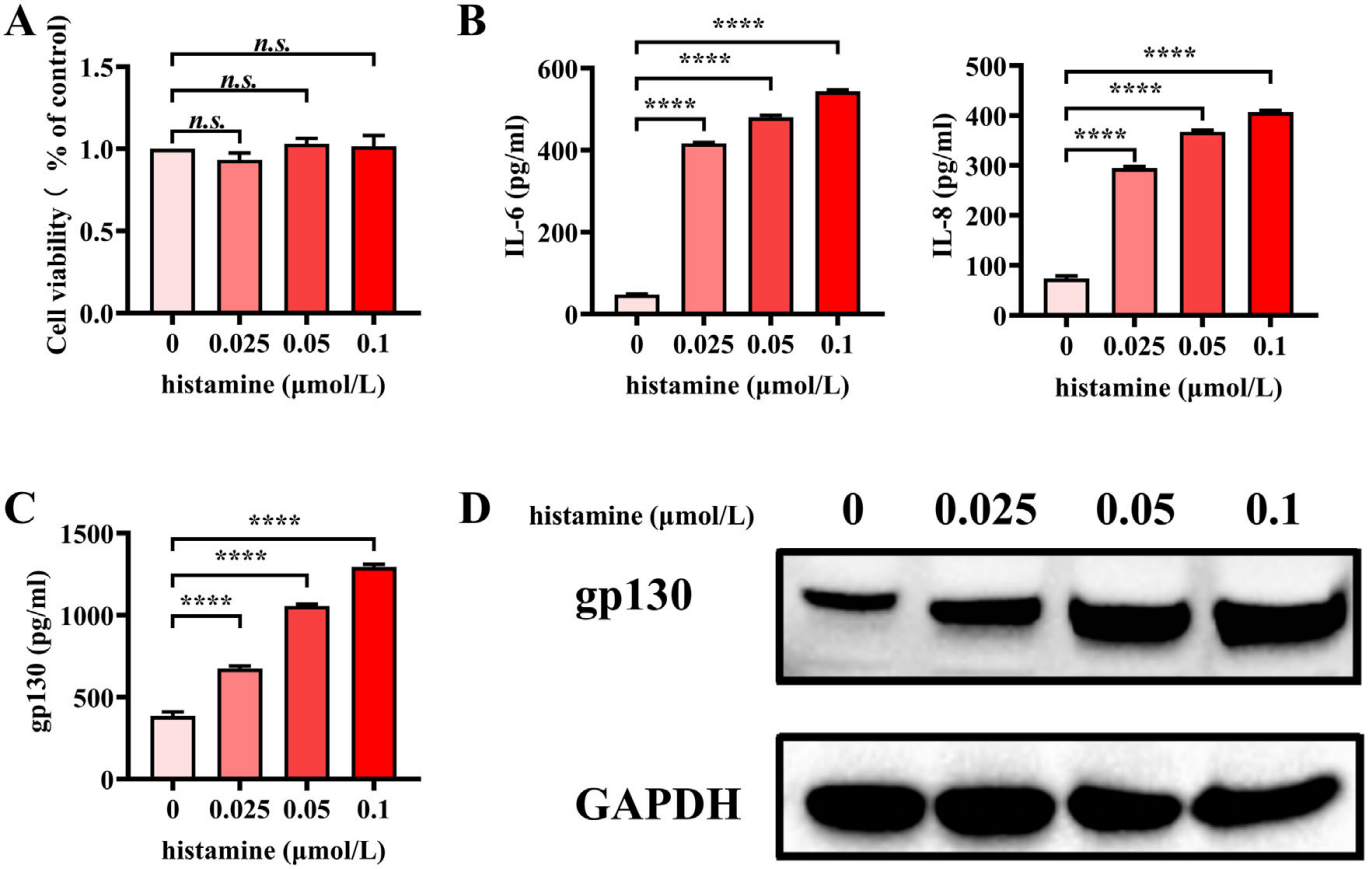
Expression of gp130 in histamine-stimulated human conjunctival epithelial cells (HConEpiCs). (**A**) Cell viability was assessed using the MTT assay. (**B,** **C**) Concentrations of cytokines in the cell supernatant, as detected via enzyme-linked immunosorbent assay (ELISA). (**D**) Expression levels of gp130 as determined using western blot analysis. GAPDH, glyceraldehyde 3-phosphate dehydrogenase. ****P < 0.0001.

Figure 4A shows that after treatment with histamine, cell viability was unchanged. These results indicated that the prescribed concentrations of histamine were not toxic to HConEpiCs. Previous research has shown that elevated IL-6 and IL-8 levels are crucial for AC development.^34^^–^^36^ The secretion of IL-6 and IL-8 into the cell supernatant increased significantly after histamine stimulation (Fig. 4B). Quantification of the gp130 concentration in the supernatant using ELISA produced a similar result, revealing increased gp130 production by histamine-stimulated HConEpiCs (Fig. 4C). Compared to control cells, histamine-stimulated HConEpiCs cells exhibited significantly increased expression of gp130 in a dose-dependent manner (Fig. 4D).

### Gp130 Participates in JAK2/STAT3 Activation in AC

**Figure 5.**
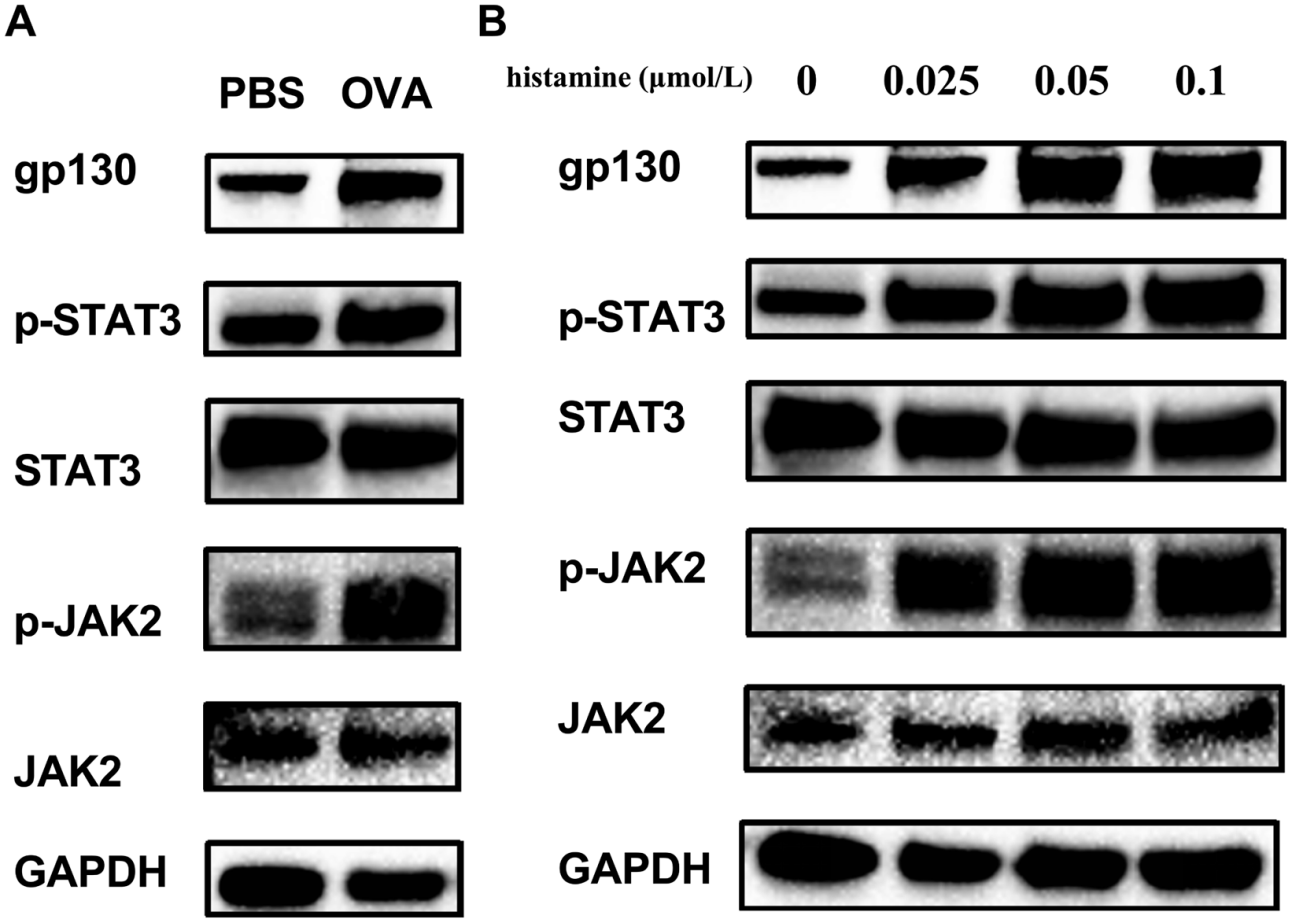
Involvement of gp130 in Janus kinase-signal transducer and activator of transcription (JAK/STAT3) activation in allergic conjunctivitis (AC), showing the expression levels of JAK2, p-JAK2, STAT3, and p-STAT3 determined using western blot analysis. (**A**) Mouse conjunctival tissue. Phosphate-buffered saline, PBS; OVA, ovalbumin-induced mouse group; GAPDH, glyceraldehyde 3-phosphate dehydrogenase. (**B**) Histamine-stimulated human conjunctival epithelial cells (HConEpiCs). PBS, normal mouse group. OVA, ovalbumin-induced mouse group.

P-STAT3 and p-JAK2 were upregulated in the conjunctival tissue of OVA-induced mice compared with normal conjunctival tissue (Fig. 5A). Furthermore, as shown in Figure 5B, histamine treatment significantly promoted the phosphorylation of STAT3 and JAK2 in HConEpiCs. Taken together, these results suggest that elevated Gp130/JAK/STAT3 signaling may provide essential contributions to AC.

### Inhibition of gp130 Phosphorylation Alleviates Ocular Surface Inflammation in Mice

**Figure 6.**
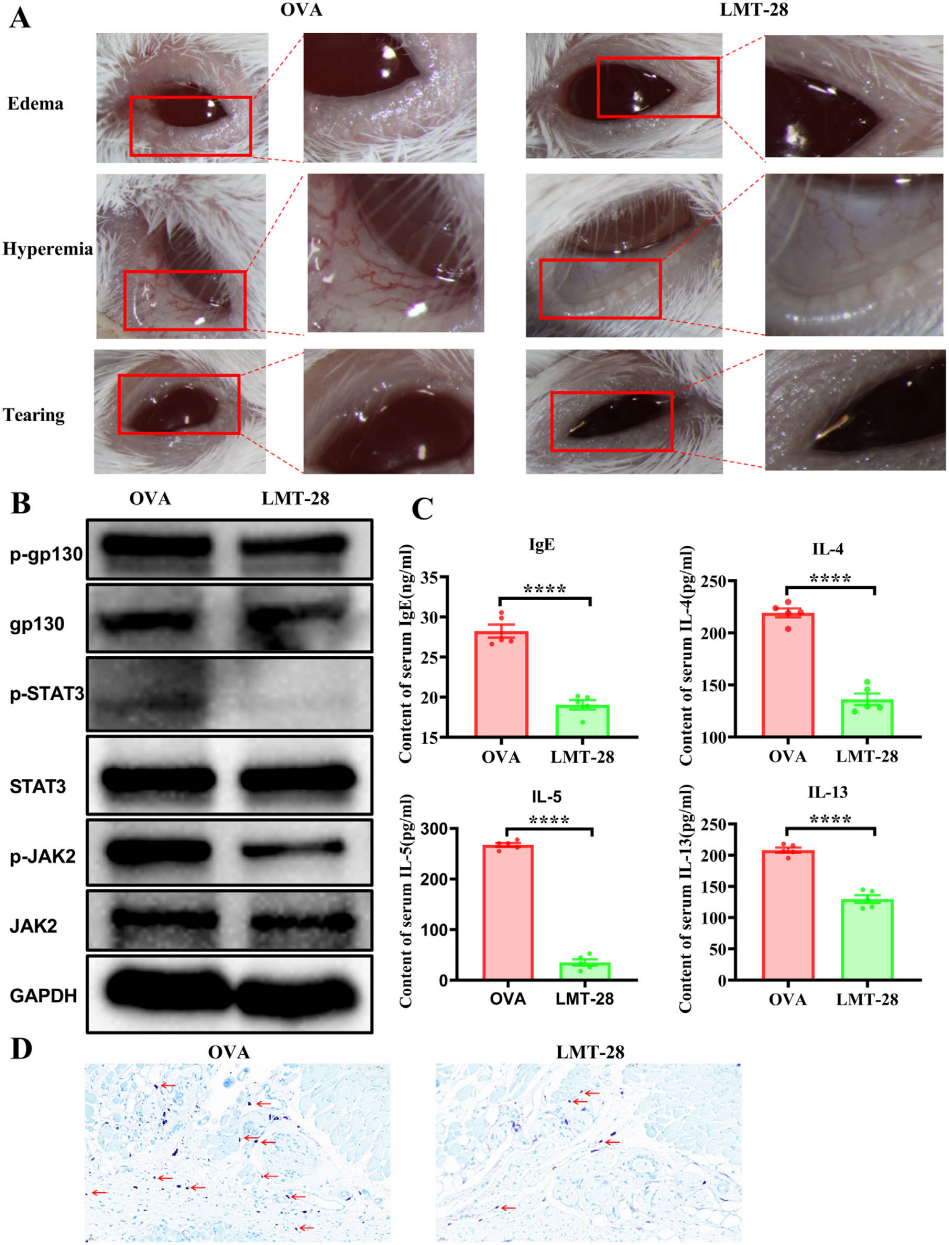
Inhibition of gp130 phosphorylation alleviates ocular surface inflammation in mice. (**A**) Ocular surface symptoms in mice. OVA, ovalbumin-induced mouse group. LMT-28, LMT-28 treated mouse group. (**B**) Protein levels of p-gp130, p-STAT3, and p-JAK2 in conjunctival tissue were detected using Western blot analysis; glyceraldehyde 3-phosphate dehydrogenase (GAPDH) was used as the loading control. OVA, ovalbumin-induced mouse group. LMT-28, LMT-28 treated mouse group. (**C**) Concentrations of IgE, IL-4, IL-5 and IL-13 in the mice serum were detected via ELISA. OVA, ovalbumin-induced mouse group, *n* = 5. LMT-28, LMT-28 treated mouse group, *n* = 5, ****P < 0.0001. (**D**) Mast cells in conjunctival tissue were detected by toluidine blue staining (40×). *Arrows* indicate the toluidine blue-stained mast cells.

To further understand the role of gp130 in AC, we applied LMT-28 to the ocular surface of mice 3 times daily to inhibit gp130 phosphorylation. As shown in Figure 6A, ocular surface inflammation was significantly relieved in LMT-28-treated mice (LMT-28 group) compared to OVA-challenged mice. Specifically, relatively minor eyelid redness and swelling, conjunctival congestion and edema, and tearing were observed. P-STAT3 and p-JAK2 were downregulated in the conjunctival tissue of LMT-28-treated mice compared with that of OVA-challenged mice (Fig. 6B). Serum IgE, IL-4, IL-5, and IL-13 level was determined in mice and the results displayed a low level of IgE, IL-4, IL-5, and IL-13 in LMT-28 treated mice (Fig. 6C). Inflammatory infiltration of mast cells was decreased by toluidine blue staining (Fig. 6D).

## Discussion

Globally, the AC prevalence has increased over the past decades.^37^ Work and educational productivity, as well as overall quality of life, are adversely affected by AC. In health and welfare policies, the issues caused by AC can have enormous economic costs.^38^ However, the pathogenesis of AC is not yet well understood. Identifying the molecular and cellular mechanisms associated with AC would help to further elucidate the mechanisms underlying the pathological transformation and pathogenesis of AC. Therefore, the effects of gp130 related to AC were explored by expression detection.

In this study, we hypothesized that gp130 is essential for AC, as gp130 levels in tears and serum are elevated in patients with AC; hence, the concentration could reflect the prognosis and severity of AC. In a murine model, gp130 levels were increased in the OVA-induced mouse conjunctiva. Moreover, both the expression and secretion of gp130 were significantly increased in a dose-dependent manner in histamine-stimulated HConEpiCs compared to control cells. As researchers look for novel candidate biomarkers to improve the management and outcome of AC, gp130 is emerging as a signal transmitter with potential value as a predictor. Gp130 seems to be a positive independent prognostic marker, with higher expression levels associated with a worse prognosis.

Gp130 in both tears and serum was found to be positively correlated with increasing itch scores in the present study. Gp130 in tears may contribute to the aggravation of ocular symptoms. Inflammation is linked to the most well-known ligands: cytokines.^13^ Gp130 plays a direct role in regulating cellular recruitment to sites of local inflammation, according to the literature.^39^^,^^40^ This suggests that gp130 plays a major role in balancing pro- and anti-inflammatory responses during inflammation.^41^ In tears and serum, total IgE was positively correlated with gp130. The central roles of IgE in AC development and manifestation are increasingly evident. The total serum IgE level reflects the immune response throughout the body.

Type 1 hypersensitivity reactions, such as AC, are mediated by histamine.^33^ A major mast cell secretory product, histamine causes conjunctival inflammation mediated by invading inflammatory cells, inducing redness, itching, and edema.^42^ IL-6 and IL-8 are released by the conjunctival epithelium. In the development of allergic diseases, histamine is known to contribute to the expression of IL-8 and IL-6.^33^^,^^43^ Here, histamine was also found to induce the expression of IL-6 and IL-8. This finding suggests that the state of conjunctival cells in AC can be recapitulated by stimulating cells with histamine in vitro. Similarly, the present research demonstrated that histamine increased gp130 secretion by HConEpiCs. To explore the underlying mechanisms and develop an efficient treatment, an OVA-induced mouse model was generated; compared with normal mice, model mice showed increased gp130 levels in conjunctival tissue, consistent with the clinical results. The in vivo and in vitro experimental results presented here show that gp130 plays an important biological function in AC, which warrants further exploration.

There are three intracellular signaling pathways activated by all cytokines: the JAK/STAT, MAPK/ERK, and PI3K pathways.^44^ Cytokines in the IL-6 family mainly activate STAT3.^45^ Moreover, among the JAK family members, JAK1, JAK2, and TYK2 were correlated with IL-6 family receptors. Gp130 is a ubiquitous β-receptor for all IL-6 family cytokines.^46^ The activation of gp130 also activates other JAK-dependent pathways in addition to STAT signaling. JAK2 is activated by extracellular ligands (including those in the IL-6 family). In turn, STAT3 translocates into the nucleus to transactivate target genes upon recruitment and activation of JAK2.^47^ OVA challenge upregulated the expression of the proteins gp130, JAK2, and STAT3 in conjunctival tissue. Similarly, histamine-treated HConEpiCs displayed activation of the gp130/JAK2/STAT3 signaling pathway.

LMT-28 is a derivative from oxazolidinone, which is the first synthesized active IL-6 inhibitor that functions at direct binding to gp130. LMT-28 shows low toxicity and selectively inhibits IL-6 activated phosphorylation of STAT3, JAK2, and gp130. LMT-28 interacts directly with gp130, inhibits IL-6/IL-6Rα complex binding to gp130, and possibly inhibits gp130 homodimer-induced signaling.^19^ Yeon-Hwa Park and colleagues confirmed that LMT-28 diminished IL-6-mediated gp130, STAT3, and ERK signaling.^48^ Particularly, a study conducted in Korea found that LMT-28 inhibited phosphorylation of GP130, STAT3, and ERK induced by Hyper-IL-6 in human fibroblast-like synoviocytes.^49^ Therefore, in our study, we chose LMT-28 to inhibit the activity of gp130. We also found that the IL-6 inhibitor LMT-28 could suppress AC inflammation in OVA-challenged mice, showing potential for AC treatment. All of these lines of evidence suggest that the gp130/JAK2/STAT3 pathway could have a significant impact on the occurrence and development of AC.

## Conclusions

In summary, this study reveals that gp130 may play an important role in AC through the gp130/JAK2/STAT3 pathway. Inhibition of gp130 phosphorylation alleviates ocular surface inflammation in mice, which may be a potential treatment approach for AC.

## Supplementary Material

Supplement 1
