# Supplementary material for: Gp130 Promotes Inflammation via the STAT3/JAK2 Pathway in Allergic Conjunctivitis
Source: Invest Ophthalmol Vis Sci. 2023 Apr 6;64(4):5. doi: 10.1167/iovs.64.4.5 (PMC10082384; doi:10.1167/iovs.64.4.5)
Supplement: Supplement 1 [file iovs-64-4-5_s001.pdf]

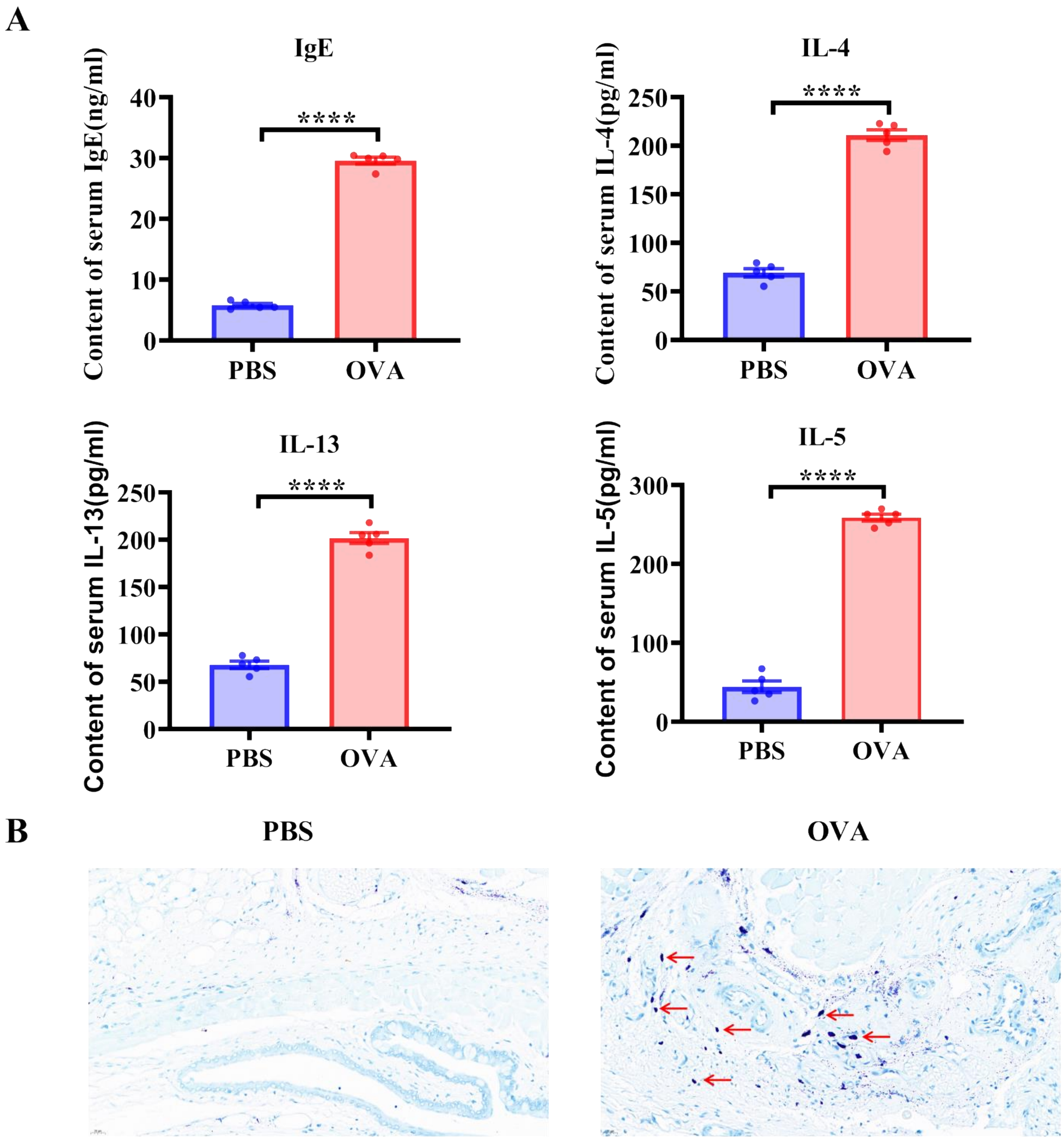

Fig. S1. AC was successfully induced in the mice by OVA treatment. **A** Concentrations of IgE, IL-4, IL-5 and IL-13 in the mice serum were detected via ELISA. PBS, normal mouse group, n=5. OVA, ovalbumin-induced mouse group, n=5. **B** Mast cells in conjunctival tissue were detected by toluidine blue staining (40×). Arrows indicate the toluidine blue-stained mast cells. ****P<0.0001
